# Augmented Flow-Induced Outward Remodelling Occurs with Ageing in Mice

**DOI:** 10.3390/ijms262110274

**Published:** 2025-10-22

**Authors:** Bethan Brown, Helen Williams, Samson Egbulonu, Andrew Bond, Jason Johnson, Sarah George

**Affiliations:** Translational Health Sciences, Bristol Medical School, University of Bristol, Level 7 Bristol Royal Infirmary, Bristol BS2 8HW, UK; bethan.a.brown@gmail.com (B.B.); samson.egbulonu@yahoo.com (S.E.); andrew.bond@bristol.ac.uk (A.B.); jason.l.johnson@bristol.ac.uk (J.J.); s.j.george@bristol.ac.uk (S.G.)

**Keywords:** ageing, artery, flow remodelling, cardiovascular disease

## Abstract

Outward remodelling of arteries is a feature of cardiovascular pathologies such as atherosclerosis and aneurysm, so a greater understanding of the processes involved in remodelling may aid the development of improved therapies for patients. As ageing increases the risk of atherosclerosis and aneurysmal disease, it was therefore hypothesised that ageing affects arterial remodelling and thereby contributes to these diseases. To test this hypothesis, we compared right carotid artery remodelling in young (2 months, n = 13) and old (18–20 months, n = 13) mice resulting from increased blood flow after ligation of the left carotid artery. The media area, thickness, collagen content and α-SM-actin content per cell of control right carotid arteries from old mice were significantly greater than observed in young mice. Positive remodelling was observed in the carotid arteries of both old and young mice 21 days after ligation of the left carotid artery. However, arteries from old mice had a significantly larger increase in lumen size and reduction in media area, thickness and α-SM-actin content per cell compared to young arteries, indicative of augmented positive remodelling in arteries from old mice. Remodelling was associated with significantly increased MMP-2 protein in arteries from young mice, but this was not observed in arteries from old mice. This study demonstrates that the extent of positive remodelling of carotid arteries is greater in old mice than in young mice and results in a potentially less resilient medial layer with decreased α-SM-actin content per cell, elastin and collagen that may promote atherosclerosis and aneurysm formation.

## 1. Introduction

Atherosclerosis and aneurysm formation represent a significant morbidity and mortality burden, particularly in Western countries. The British Heart Foundation state that 1 in 12 people globally suffer from cardiovascular disease, and that this number has doubled between 1993 and 2021 (bhf.org.uk). Outward or expansive remodelling of the blood vessel wall is involved in both atherosclerosis and aneurysm. In the case of atherosclerosis, compensatory enlargement of the blood vessel, known as the Glagov phenomenon [1], occurs to prevent loss of perfusion in early stenosis [2]. However, despite limiting initial stenosis, outward remodelling is associated with an inflammatory and unstable plaque phenotype [3,4,5,6] and a higher incidence of major adverse cardiac events [7], suggesting that in the long term, outward remodelling may be detrimental for disease outcome. Remodelling of the vascular wall also occurs in aneurysm formation, characterised by dilations in the arterial wall which expand until weakness results in rupture and haemorrhage and often death [8]. Similar to atherosclerosis, aneurysm pathology is complex, involving immune cell infiltration, inflammation, oxidative stress, protease-mediated collagen and elastin degradation and VSMC apoptosis [9,10,11].These processes result in restructuring of the vascular wall and dilation. It is therefore possible that further understanding of remodelling of the vascular wall may lead to the identification of novel targets to reduce vessel expansion and associated negative outcomes in both aneurysm and atherosclerosis.

Ageing is a well-known risk factor for both atherosclerosis and aneurysm formation. As we age, our vasculature sustains a range of complex changes culminating in thickening and stiffening of the arterial wall, notably mirroring the structural and cellular alterations observed in vascular disease [12,13,14]. More specifically, ageing has been shown to advance the progression of atherosclerosis in animal models of ageing [15,16] or senescence [17,18] and patients with the accelerated ageing syndrome Hutchinson–Gilford progeria syndrome [19,20]. Similarly, ageing has been identified as a prominent risk factor for abdominal aortic aneurysm formation in a combined cohort of over 125,000 patients [21,22]. Therefore, it is important to appreciate the effect of age on outward remodelling to optimise the impact of cardiovascular intervention in elderly patients.

In the current study, we employ a murine model of left carotid artery ligation to assess the effect of ageing on outward remodelling of the contralateral carotid artery in response to heightened blood flow (details of the model have been reported previously [23]). Previous studies have shown that after carotid artery ligation, blood flow is quickly increased in the contralateral carotid artery and is maintained for the period of ligation with a 60% increase in blood flow in the right carotid mouse artery following ligation of the left carotid [24,25]. Although endothelium-dependent relaxation of vascular tone can regulate acute increases in blood flow [26,27], evidence suggests that in response to a prolonged rise in flow, arteries undergo outward remodelling involving structural changes to the vascular wall that expand the lumen and normalise wall shear stress [28]. This outward remodelling has been reported in the contralateral carotid artery following unilateral carotid artery ligation in both rats and mice [24,25,29,30,31]. However, the effect of age on this process in carotid arteries has not been described.

Evidence suggests that although the blood flow increases within minutes, adaptive changes to the vessel structure occur over days to weeks and involve modification of both vascular cell behaviour and protease expression [24,31]. Studies investigating flow augmentation in the carotid arteries have demonstrated pivotal roles for endothelium-dependent nitric oxide (NO) release and subsequent matrix-degrading metalloproteinase (MMP) activation in outward remodelling. It was then shown that NO was necessary for activation of the protease MMP-2 and that MMPs were necessary for elastin degradation, allowing outward remodelling [32]. This essential role for MMPs has since been reported by multiple groups [25,33,34], including the gelatinases MMP-2 and MMP-9, which are both expressed following flow augmentation [24,25,29,32]. The abundance of MMPs within the arterial wall has been shown to increase with age, and many studies have focussed on MMP-2 [35,36,37,38]. Li and co-workers observed co-localisation of MMP-2 with degraded medial elastin in the ageing rat aorta, implying MMP-2 involvement in ECM degradation in aged arteries [35]. Wang and colleagues reported that long-term in vivo treatment of rats with a broad-spectrum MMP inhibitor prevented age-related elastin degradation and collagen accumulation compared to controls [38]. As MMP-2 has been shown to regulate outward remodelling in response to high blood flow, it can be hypothesised that arterial remodelling in response to increased blood flow differs in old and young mice and may be regulated in part by MMP-2. This study therefore compared the remodelling of the right carotid artery in response to the increased blood flow caused by ligation of the left carotid in old and young mice. We quantified the morphological changes that took place in the right carotid artery and examined levels of MMP-2 and structural proteins involved in remodelling in old and young mice.

## 2. Results

### 2.1. Remodelling of Right Carotid Artery Was Augmented in Old Mice Compare to Young

**Figure 1:**
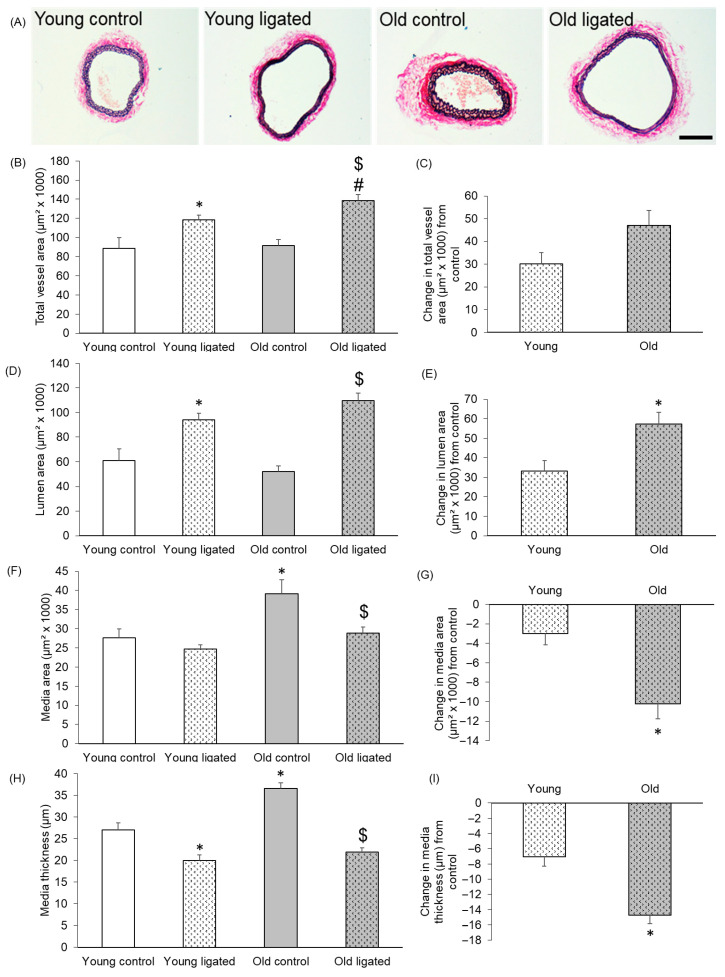
Histological comparison of the right carotid artery before and 21 days after ligation of the left carotid artery in young and old mice. (**A**) Representative images of EVG stained sections of right carotid arteries from young and old mice 21 days after ligation of the left carotid artery or from unligated controls. The scale bar represents 100 μm and applies to all images. The total vessel area (**B**), lumen area (**D**) of right carotid arteries was measured on EVG stained sections. The media area (**F**) of right carotid arteries was calculated by subtraction of the lumen area from the total vessel area. Media thickness (**H**) in right carotid arteries was measured at eight approximately equidistant points around the vessel wall of EVG stained sections and then averaged to give a mean media thickness for each vessel. For all graphs * indicates *p* < 0.05 vs. young control, $ indicates *p* < 0.05 vs. old control, # indicates *p* < 0.05 vs. young ligated, ANOVA and Student Newman-Keuls post hoc test, n = 4 controls and n = 13 young, n = 13 old, ligated. The change in the total vessel area (**C**), lumen area (**E**), media area (**G**) and medial thickness (**I**) of right carotid arteries following let carotid artery ligation was calculated for each age group by subtracting the average parameter measurement of the unligated arteries from that of the ligated group. * indicates *p* < 0.05 vs. young, unpaired Student’s *t*-test, n = 13 young, n = 13 old.

EVG-stained transverse sections of right carotid arteries from young and old mice are shown in Figure 1A. It was observed that although the carotid artery total vessel and lumen areas were comparable in old and young mice, the media area and thickness was significantly greater in arteries from old mice than young mice (Figure 1). Total vessel area of the right carotid artery was significantly increased by elevated blood flow in both young and old mice (Figure 1B). Although there was no difference in the total vessel area of control arteries with age, the resultant total area following high blood flow was significantly greater in old mice (Figure 1B). Further analysis was performed to calculate the effect of ligation on contralateral arteries from each age group. A greater change in total vessel area was detected in old mice following elevated blood flow, but this did not reach significance (*p* = 0.053) (Figure 1C). The lumen area of the right carotid artery was significantly increased after ligation in both young and old mice to approximately the same area (Figure 1D). However, further analysis demonstrated that the change in lumen area was significantly greater in arteries from old compared to young mice (Figure 1E). Together, this suggests that although enhanced blood flow increased the right carotid artery lumen area in mice of both ages, this expansion was greater in older mice. In young mice, ligation of the left carotid artery did not significantly affect the right carotid media area, whereas in old mice, the medial area was significantly reduced (Figure 1F,G). A significant reduction in media thickness was detected in both young and old mice (Figure 1H); however, the reduction was significantly greater in arteries from old mice (Figure 1I). Together, the data demonstrate that control carotid arteries from old mice have larger, thicker media than their younger counterparts. Ligation of the left carotid artery induced thinning of the media in right carotids at both ages, but the extent of this remodelling was augmented with age. Subsequently, the resultant area and thickness of the media was comparable in young and old right carotid arteries 21 days after ligation (Figure 1F,H).

### 2.2. Medial Cell Number and Density, Proliferation and Apoptosis Did Not Differ in Old and Young Mice

**Figure 2:**
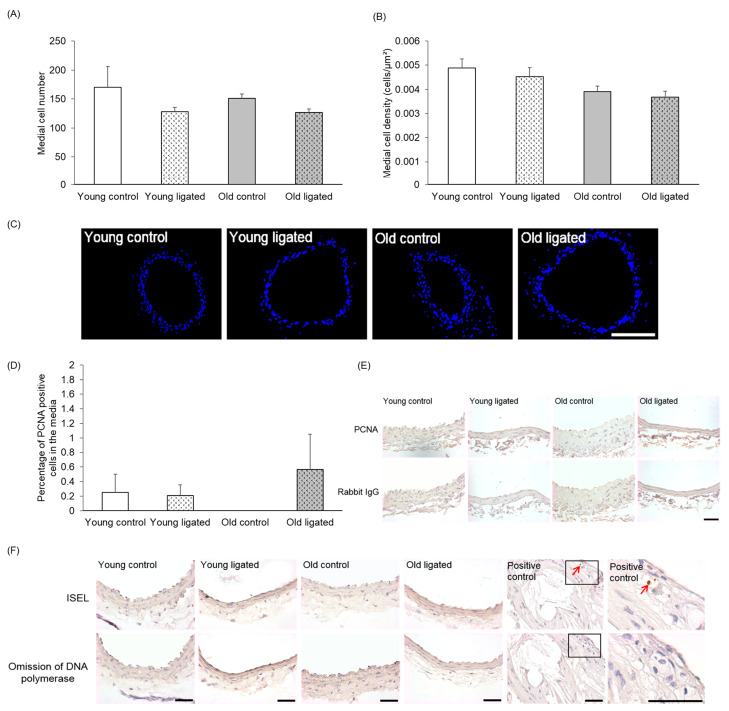
Quantification of medial cell number, density, proliferation and apoptosis in the right carotid artery before and 21 days after ligation of the left carotid artery in young and old mice. Young and old mice were subjected to ligation of the left carotid artery for 21 days. Animals with unligated arteries served as controls. The number of medial cells in right carotid arteries was counted in DAPI stained sections (**A**), Medial cell density in right carotid arteries was calculated by normalisation of medial cell counts to medial area (**B**). No significant differences were observed, ANOVA and Student Newman-Keuls post hoc test, n = 4 controls and n = 13 ligated in each age group. (**C**) Representative images of DAPI stained sections (blue nuclei) of right carotid areres from young and old mice. The scale bar represents 100 μm. The number of proliferating medial cells in right carotid arteries were counted on PCNA stained sections and expressed as a percentage of the total media cell number (**D**). No significant differences were observed, ANOVA and Student Newman-Keuls post hoc test. n = 4 controls and n = 13 young and n = 13 old ligated. (**E**) Representative of PCNA immunohistochemistry, PCNA positive cells have brown nuclei and PCNA negative cells have haematoxylin-stained blue nuclei. Non-immune rabbit lgG was employed as a negative control. (**F**) Representative images of ISEL. ISEL positive cells (brown shrunken nuclei indicated by red arrows) were not observed in any right carotid artery examined (n = 3 in each group) but were present in the positive control of a murine brachiocephalic artery atherosclerotic plaque. ISEL negative cells have haematoxylin-stained blue nuclei. Omission of DNA polymerase was employed as a negative control. The scale bar represents 50 μm in images E&F.

Neither medial cell number nor density significantly differed with age or after ligation of the left carotid artery (Figure 2A–C). Very low levels of medial proliferation were observed, with only six PCNA-positive cells detected in total from all right carotids (Figure 2D,E). Proliferation rates were not altered by age or after ligation of the left carotid artery (Figure 2D,E). No ISEL-positive cells were detected in right carotid arteries from any vessels (Figure 2F). The ISEL protocol was verified by inclusion of a mouse brachiocephalic artery atherosclerotic plaque section as a positive control [39].

### 2.3. α-SM-Actin Content per Medial Cell Was Enhanced with Age but Unaffected by Elevated Blood Flow

**Figure 3:**
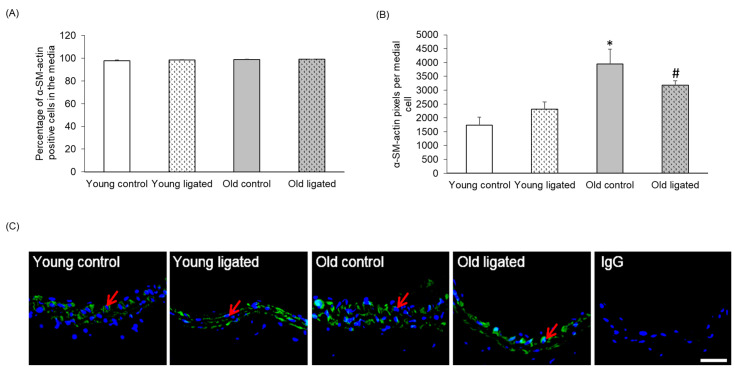
Quantification of medial α-SM-actin in the right carotid artery before and 21 days after ligation of the left carotid artery in young and old mice. Young and old mice were subjected to ligation of the let carotid artery for 21 days. Animals with unligated arteries served as controls. (**A**) Immunofluorescence was used to detect α-SM-actin. The number o α-SM-actin positive medial cells was expressed as a percentage of the total media cell number viewed. No significant differences were observed, ANOVA and Student Newman-Keuls post hoc test, n = 4 controls and n = 13 ligated in each age group. (**B**) The number of α-SM-actin pixels per medial cell in right carotid arteries was analysed by pixel analysis and normalisation to media cell number. * indicates *p* < 0.05 vs. young control, # indicates *p* < 0.05 vs. young ligated, ANOVA and Sludent Newman-keuls post hoc test, n = 4 controls and n = 13 young and n = 13 old ligated. (**C**) Representative images of immunofluorescence for α-SM-actin sections of right carotid arteries from young and old mice 21 days alter ligation of the let carotid artery or from unligated controls, Examples of α-SM-actin positive cells (green) are indicated with red arrows, Nuclei are stained with DAPI (blue), The scale bar represents 50 μm.

Almost all cells in the right carotid media were α-SM-actin-positive, and the number of VSMCs was unaffected by age or ligation of the left carotid artery (Figure 3A,C). Medial cells in control arteries from old mice had significantly more α-SM-actin per cell than young mice (Figure 3B). Elevated blood flow did not significantly affect the amount of α-SM-actin per cell in either age group, and therefore the age-associated difference was maintained following ligation (Figure 3B).

### 2.4. Medial Elastin Content Was Lower in Old Mice with Elevated Blood Flow

**Figure 4:**
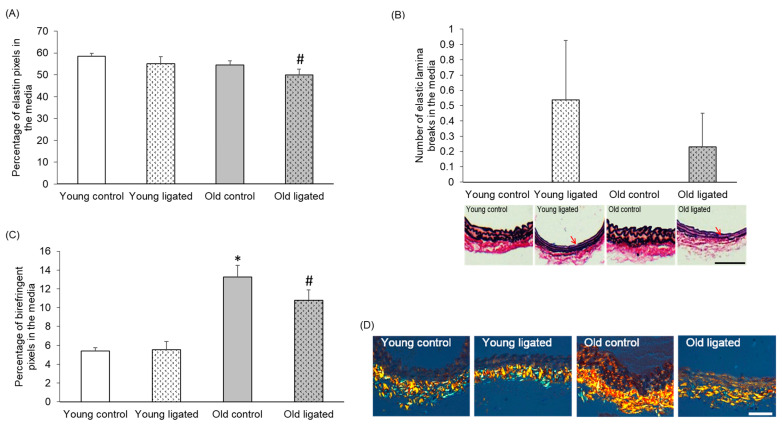
Quantification of elastin and collagen content in the right carotid artery before and 21 days after ligation of the left carotid artery in young and old mice. Young and old mice were subjected to ligation of the left carotid artery for 21 days. Animals with unligated arteries served as controls. (**A**) Medial elastin content in right carotid arteries was measured by pixel analysis of EVG stained sections and expressed as a percentage of medial area. # indicates *p* < 0.05 vs. young ligated, ANOVA and Student Newman-Keuls post hoc test, n = 4 controls and n = 13 young and n = 13 old ligated. (**B**) The number of elastic lamina breaks in the media of right carotid arteries was counted in EVG stained sections. No significant differences were observed, ANOVA and Student Newman-Keuls post hoc test, n = 4 controls and n = 13 ligated in each age group. Representative images are shown with elastic lamina breaks indicated with red arrows. (**C**) Medal collagen content in right carotid arteries was measured by pixel analysis on picrosirius red stained sections and expressed as a percentage of media area. * indicates *p* < 0.05 vs. young control, # indicates *p* < 0.05 vs. young ligated, ANOVA and Sludent Newman-Keuls post hoc test. n = 4 controls and n = 13 young and n = 13 old ligated. (**D**) Representative images of picrosirius red stained sections. Collagen appears bright orange/yellow/green when viewed under polarised right The adventitia stains strongly for collagen (near the bottom of each image), while collagen staining in the media is lighter in comparison (near the top of each image). The scale bars represent 50 μm.

The elastin content in the media was not visibly affected by age or elevated blood flow; however, a small but significant decrease in elastin content was detected between arteries from old mice after ligation of the left carotid artery compared to young mice (Figure 4A). No elastic lamina breaks were observed in arteries from control mice of either age. However, elastic lamina breaks were observed in right carotids from 5 out of 13 young and 3 out of 12 old mice that underwent ligation. Although elastic lamina breaks were only identified in arteries from ligated mice, a significant difference in the total number of breaks following elevated blood flow was not seen at either age (Figure 4B).

### 2.5. Medial Collagen Content Was Increased with Age but Unaffected by Elevated Blood Flow

Medial collagen content was significantly higher in control arteries from old mice compared to young (Figure 4C,D). Ligation of the left carotid artery had no significant effect on collagen content at either age; hence, this age-associated difference was maintained after enhanced blood flow (Figure 4C). Collagen content was greatest in the adventitia of all arteries, as previously noted by others [40].

### 2.6. MMP-2 Protein Was Increased by Elevated Blood Flow in Young, but Not Old, Mice

**Figure 5:**
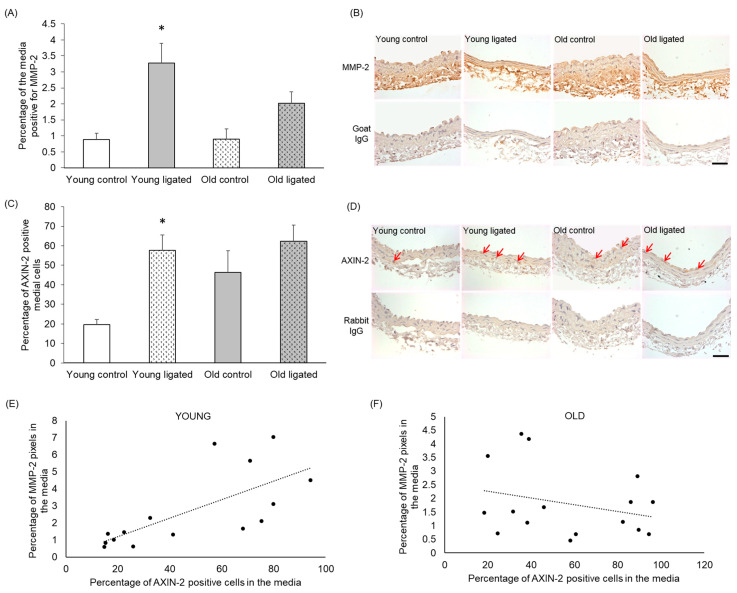
Quantification of MMP-2 and AXiN-2 in the right carotid artery before and 21 days after ligation of the left carotid artery in young and old mice. Young and old mice were subjected to ligation of the left carotid artery for 2 days, Animals with unligated arteries served as controls. (**A**) MMP-2 protein detected by immunohistochemistry in the media of right carotid arteries was quantified by pixel analysis on sections and expressed as a percentage of medial area. * indicates *p* < 0.05 vs. young contol, Kruskal-Walls test and Dunn’s multiple comparisons test, n = 4 young controls, n = 12 young ligated, n = 3 old controls, n = 13 old ligated. (**B**) Representative images of MMP-2 immunohistochemistry, MMP-2 protein is brown and haematoxylin-stained nuclei are blue. (**C**) Medial cells positive for AXiN-2 (detected by immunohistochemistry) were counted on sections of right carotid arteries and expressed as a percentage of the total medial cell number viewed, * indicates *p* < 0 05 vs. young control, ANOVA and Student Newman-Keuls post hoc test, n = 4 young controls, n = 11 young ligated, n = 4 old controls, n = 12 old ligated. (**D**) Representative images ofAXN-2immunohistochemistry with some positive cells with brown cytoplasm are indicated by red arrows. Haematoxylin-stained nuclei are blue. (**E**,**F**). The percentage of AXIN-2 positive cells and MMP-2 protein in the media of right carotid arteries were plotted and correlation was assessed for young (**E**) and old (**F**) vessels (n = 15 young, n = 16 old. A linear trendline is shown on each graph. Significant correlation was detected in young arteries (r^2^ = 0.4891) but not old arteries (r^2^ = 0.07847). The scale bars represent 50 μm.

Sections were stained using an antibody that detected both pro- and active forms of MMP-2 [41]. There was no significant difference in the percentage of the media positive for MMP-2 in arteries from young and old control mice (Figure 5A,B). However significantly greater abundance of MMP-2 was observed in the right carotid of young mice subjected to ligation of the left carotid artery, while no significant increase was observed in old mice (Figure 5A,B).

### 2.7. AXIN-2-Positive Cells Increased with Elevated Blood Flow in Young, but Not Old, Mice

To determine whether Wnt β-catenin signalling was activated in right carotid arteries following elevated blood flow, right carotids were stained for the β-catenin/TCF responsive gene AXIN-2 [42,43,44]. The percentage of AXIN-2-positive medial cells appeared to be increased with age, although this was not significant with n = 4 (Figure 5C,D). In young arteries, the percentage of AXIN-2-positive medial cells was increased following high blood flow, whereas in old arteries, no significant change in the number of AXIN-2-positive cells was observed (Figure 5C,D).

### 2.8. Medial AXIN-2 and MMP-2 Abundance Correlated in Young, but Not Old, Mice

Correlation analysis was performed between AXIN-2 and MMP-2 expression using both control and ligated mice in both age groups. In arteries from young mice, there was a significant correlation between the percentage of AXIN-2-positive cells and MMP-2 content in the media (Figure 5E, r^2^ = 0.4891), whereas in old arteries, a correlation was not observed (Figure 5F, r^2^ = 0.07847).

### 2.9. Reduction in Adventitial Collagen Following Elevated Blood Flow Was Greater with Age

**Figure 6:**
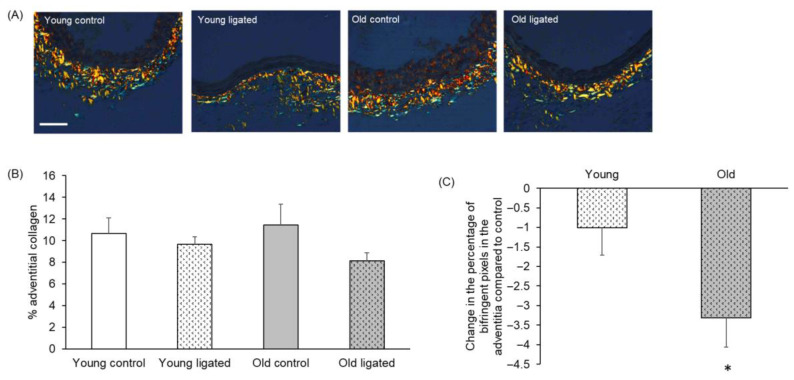
Quantification of adventitial collagen in the right carotid artery before and 21 days after ligation of the left carotid artery in young and old mice. (**A**) Representative images of picrosirius red stained sections of right carotid arteries. The scale bar represents 50 μm and applies to all images. (**B**) The percentage of the adventitial staining positive for birefringent collagen was measured. No significant differences, ANOVA and Student Newman-Keuls post hoc test, n = 4 controls and n = 13 ligated in each age group. (**C**) Change in the percentage of adventitial collagen of right carotid arteries following left carotid artery ligation was calculated for each age group by subtracting the average percentage of adventitial collagen of the unligated areres from the percentage of adventitial collagen of each member of the ligated group. * indicates *p* < 0.05 vs. young, unpaired Student’s *t*-test, n = 13 young and n = 13 old ligated.

Adventitial extracellular matrix collagen in right carotid arteries was quantified to determine whether changes in the adventitial layer could contribute to the differential outward remodelling observed with age. Figure 6B shows that although the percentage of collagen in the adventitia did not change to a significant degree with age or ligation of the left carotid artery, a significantly greater loss of adventitial collagen was observed in arteries from old compared to young mice (Figure 6C). 

### 2.10. Macrophages Were Not Detected 21 Days After Ligation

As macrophage infiltration has been shown to have a role in outward remodelling [24], right carotid arteries were stained for the pan-macrophage marker CD68 to assess changes in macrophage infiltration between groups. However, as shown in Supplementary Figure S1, CD68 was not detected in right carotid arteries from any group. As a positive control, the presence of CD68 staining was confirmed in an atherosclerotic control artery.

## 3. Discussion

### 3.1. Comparison of Right Carotid Arteries from Young and Old Mice

Total vessel area and lumen area were not affected by age, but there was a significant increase in both medial area and thickness in arteries from old mice. This observation supports previous reports that medial thickness increases with age and is regarded as a sign of vascular ageing in mice [45,46], rats [35,47,48], non-human primates [36,49] and humans [37]. The number and density of medial cells did not change, and negligible levels of proliferation and apoptosis were observed in all arteries. The number of medial cells positive for α-SM-actin was unchanged with age, and the negligible proliferative rates in the carotids suggests that medial VSMCs maintain a quiescent phenotype in aged murine arteries; however, assessment of other phenotypic markers would be necessary to confirm this. A significant increase in α-SM-actin per medial cell was observed with age, agreeing with a report studying monkey VSMCs [50]. It is possible that this increase in α-SM-actin may be due to an increase in cell size, which would explain the increased medial thickness and area with age, or simply increased expression of α-SM-actin within each VSMC.

Medial collagen was significantly augmented with age, which may contribute to the observed increase in medial area and thickness observed in old mice and may render the artery stiffer/less compliant. This corroborates previous observations that increased medial collagen occurred in the thoracic aorta of aged mice [51,52] and humans [32]. There are differing reports by other studies regarding whether elastin declines with age or is at a consistent level but with a reduction in organisation over time [53]. In our study, no difference in medial elastin content was observed in ageing arteries, and no elastic lamina breaks were seen in the arteries of control mice.

There was no significant difference in medial cells expressing MMP-2 or AXIN-2 in control arteries with age. AXIN-2 levels indicate activation of the Wnt β-catenin pathway, as AXIN-2 is an essential component of canonical Wnt signalling [54]; therefore, no change in AXIN-2 indicates that there is no significant change in β-catenin/TCF signalling with age. We were surprised not to see an increase in MMP-2 with age as this has been reported in mice [55,56] and humans [57]; however, the data reported in all these studies show increases with age that are quite subtle, and there is very large variability of data, meaning that these studies required very large n numbers to achieve significance. It is therefore likely that our study was underpowered to observe changes in MMP-2, particularly as we were measuring levels in the vessel wall, rather than in the plasma. Marchand and colleagues have reported increased β-catenin and expression of downstream genes, including CCN4/WISP-1 and versican, in mammary arteries of elderly compared to middle-aged patients [58]. Although we saw a slight trend towards increased AXIN-2 expression, this was not significant, so as with MMP-2, it is possible that the changes in the vessel wall were not sufficient to reach significance, or that in this model the pathway is not altered with age.

### 3.2. Remodelling of the Right Carotid Artery in Response to Elevated Blood Flow in Young Mice

Left carotid artery ligation led to an increase in both total vessel and lumen area and a reduction in medial thickness in the contralateral right carotid artery, corroborating previous reports [25,29]. The number and density of cells in the media of the right carotid artery did not change, and only negligible levels of proliferation and apoptosis were detected. This finding agrees with a previous study which reported the absence of proliferation in the right carotid artery up to 4 weeks after partial left carotid artery ligation in mice [59]. Almost all medial cells stained positive for α-SM-actin, indicating no or limited phenotypic changes or cell infiltration. Neither medial elastin nor collagen content was affected by elevated blood flow, agreeing with a previous study which reported no change in collagen density following carotid artery ligation in mice [60]. Elastic lamina breaks were detected in 5 out of 13 arteries from ligated young mice, while none were detected in controls, in agreement with previous reports of internal elastic lamina fragmentation in carotid arteries following high blood flow that were dependent on MMPs degrading the elastin [32]. Our finding of increased MMP-2 expression with increased blood flow suggests MMP-2 may be acting in a similar way in our model and that this may occur via the canonical Wnt β-catenin/TCF signalling pathway and AXIN-2, as we also observed an increase in AXIN-2 positive medial cells in the right carotid artery following elevated blood flow, and in young mice, there was a correlation between MMP-2 and AXIN-2 levels.

### 3.3. Remodelling of the Right Carotid Artery in Response to Elevated Blood Flow in Old Mice

Left carotid artery ligation significantly increased the total vessel area and lumen area of the right carotid artery and reduced the media thickness in both young and old mice. However, the extent of these changes was exaggerated with age. In addition to this, a reduction in media area was only observed in right carotid arteries from old mice following ligation. This suggests that although carotid arteries from old mice have a thicker and larger media than their younger counterparts; following an increase in blood flow, medial remodelling is exaggerated in older arteries, resulting in the area and thickness of the media being comparable in young and old right carotid arteries 21 days after ligation. This finding contrasts with a study in male rats where outward remodelling of the mesenteric artery in response to increased flow was reduced in 2-year-old rats compared to 3-month-old rats [61]. This group also showed that when they did the same experiment in female rats, age had no effect on the ability to remodel, and the lack of ageing effect on female rats was due to the signalling via the oestrogen receptor alpha, as knockdown of this receptor produced results comparable to those seen in male mice. The discrepancy seen between our study on male mice compared to that of Dumont et al. using male rats may reflect species differences, as has previously been reported for VSMC behaviour with ageing (see review by Monk and George [62]), the degree of ageing of the animals used or the difference in flow characteristics between the models. Our data appear more similar to the situation in humans, where aneurysm propensity increases with age, and the stiffening of vessels leaves them more vulnerable to loss of structural integrity and rupture.

The changes to the lumen and media do not appear to be related to either medial cell number, α-SM-actin expression, proliferation or apoptosis, as these did not change with age. We observed a statistically significant, but only about 5%, reduction in medial elastin, but it is unclear if this is large enough to be physiologically relevant. Other studies have reported either a decrease in elastin content with age or stable overall elastin levels with a reduction in elastin organisation [53]. More analysis would be required to know if there was a difference in elastin organisation in our study, but as we saw no difference in the number of elastic lamina breaks, this suggests no significant changes in elastin integrity in our model. There was also no change in medial collagen following ligation of the left carotid artery. There was, however, more loss of adventitial collagen in the carotids from old mice in response to increased blood flow. It is possible that loss of this structural protein from around the carotid leads to increased remodelling capacity and less resilience of the artery. This is supported by the observation that reorganisation of adventitial collagen was associated with the growth of collateral arteries in a hindlimb ischaemia model [62], and mice deficient in collagen III are more susceptible to abdominal aortic aneurysms [63].

The upregulation in MMP-2 and AXIN-2 seen in response to increased flow in young mice was lost in aged mice. It is possible that in old arteries, upregulation of MMP-2 and AXIN-2 occurred earlier than 21 days, is too variable to detect significant changes in our study, or that a different pathway is responsible for vessel remodelling in ageing. We did not detect macrophages in our tissue sections of right carotid arteries. Although macrophages are thought to be recruited in the initial inflammatory phase that occurs in response to increased flow [64], it is likely that at our observations at 21 days post-ligation were too late to observe this phase of the remodelling.

### 3.4. Study Limitations

We are aware that our study has limitations, which are possible areas of future work. Inflammation is critical in the early phases of remodelling [65], and known to be associated with ageing, but we were not able to detect any change in macrophages at 21 days, so study of earlier timepoints is important to see the effects of inflammation in this model. Our analysis has not allowed us to look at the smaller structural components such as the fenestrae [66]. We have also not directly studied the physical characteristics of the right carotid artery, such as its compliance or ability to contract and relax. We have also only studied this phenomenon in male mice, and more thorough analysis of the molecular mechanisms underlying this phenomenon is also needed.

## 4. Methods

### 4.1. Murine Carotid Ligation Model

Housing, care and all procedures involving mice were performed in accordance with the guidelines and regulations of the University of Bristol and the United Kingdom Home Office. The investigation conforms to the Guide for the Care and Use of Laboratory Animals published by the US National Institutes of Health (NIH Publication No. 85–23, revised 1996). Ligation of the left common carotid artery was performed in 13 young (2 months) and 13 old (18–20 months) C57BL6/J male mice as described previously [23]. Briefly, mice were anaesthetised with inhaled 3% isofluorane in oxygen; the left carotid artery was exposed and dissected from the carotid nerve before tying off proximal to the bifurcation using 5-0 silk suture. Post-operative analgesia was achieved by an IP injection of 0.1 mg/kg buprenorphine. After 21 days, mice were euthanised with an IP injection of sodium pentobarbitone (500 mg/kg), and the right carotid artery was dissected, rinsed in PBS and fixed in 10% formalin/PBS for 24 h. To provide a baseline, four young and old mice were used as unligated controls.

### 4.2. Histology

Fixed arteries were embedded in agar, processed and embedded in paraffin wax and 4 μm transverse sections cut [23]. Measurements of whole vessel area, lumen area, media area, media thickness and elastin content were performed on Elastin Van Gieson (EVG)-stained sections. Medial cell number and density were quantified using 4′,6-diamidino-2-phenylindole (DAPI). To analyse collagen content, vessels were stained with picrosirius red. Sections were incubated in picrosirius red solution (0.1% Sirius Red F3B, saturated aqueous picric acid, pH 1.8–2.2) for 90 min at room temperature and washed twice in 0.01N hydrochloric acid for 5 min.

### 4.3. Immunohistochemistry

Sections were de-waxed and rehydrated, 3% H_2_O_2_ was applied to inhibit endogenous peroxidases and sections were microwaved in 10 mM citrate buffer for antigen retrieval and blocked with 20% goat serum/PBS for 30 min before incubation with 1 μg/mL CD68 (LifeSpan, Newark, NJ, USA LS-C343891), 2.5 mg/mL MMP-2 (R&D, Minneapolis, MN, USA AF1488), 0.4 μg/mL AXIN-2 (Abcam, Cambridge, UK ab32197) and 1 μg/mL PCNA antibody (Abcam ab18197) in 1% BSA/PBS overnight at 4 °C. Non-immune rabbit IgG was used as a negative control, and sections of brachiocephalic arteries from ApoE^−/−^ mice fed a high-fat diet for 12 weeks were used as a positive control for CD68 immunohistochemistry. Biotinylated-goat-anti-rabbit IgG (DAKO, Agilent, Santa Clara, CA, USA E0432) 1:200 in 1% BSA/PBS was then applied, followed by ExtrAvidin-peroxidase (Sigma, Merck, Darmstadt, Germany E2886) 1:200 in 1% BSA/PBS, and SigmaFast 3,3′-diaminobenzidine (DAB) nuclei were counterstained with Mayer’s Haematoxylin. The Fluorescein Vector mouse-on-mouse (M.O.M) Immunodetection Kit (Vector Newark, CA, USA FMK-2201) was used to detect α-smooth muscle actin according to the kit instructions, to prevent non-specific binding of the antibody to mouse tissue, using 3.1 μg/mL of α-SM-actin antibody (Sigma A2547, Darmstadt, Germany). Omission of the primary antibody was employed as the negative control.

### 4.4. In Situ End Labelling—ISEL

In situ DNA end labelling (ISEL) was used to detect apoptosis. Sections of brachiocephalic arteries from ApoE^−/−^ mice fed a high-fat diet for 12 weeks were used as a positive control. Sections were de-waxed, rehydrated and digested with 5 μg/mL of proteinase-K diluted in Tris/EDTA buffer for 15 min to enable access to DNA breaks. Slides were washed twice in Tris/EDTA buffer then incubated with ISEL reaction buffer for 15 min to inhibit endogenous peroxidases, then incubated in 3% H_2_O_2_ for 5 min. Slides were incubated with ExtrAvidin-peroxidase diluted 1:200 in 10% FBS/PBS for 30 min then washed and incubated with DAB for 10 min, stained with Mayer’s Haematoxylin for 30 s, dehydrated, cleared and mounted in DPX.

### 4.5. Statistics

Data are presented as mean ± SEM. Normality was assessed using a Shapiro–Wilk test. For normally distributed data, *t*-tests or ANOVA with Student–Newman–Keuls post hoc tests were used. For non-normal data, the Kruskal–Wallis test and Dunn’s multiple comparisons test were used. Statistical significance was accepted as *p*  <  0.05. All experiments used biological replicates from different individuals with numbers stated in the legends.

## 5. Conclusions

Our study identified structural and compositional differences in carotid arteries with ageing and showed that flow-induced outward remodelling was exaggerated with age. This enhanced remodelling was not accompanied by changes in VSMC number, density, proliferation or apoptosis, α-SM-actin content per cell or medial elastin or collagen content. Exaggerated outward remodelling with age was however associated with failure to upregulate MMP-2 or AXIN-2, so underlying regulatory mechanisms may differ in old mice. Loss of adventitial collagen was exaggerated with age, so it is possible that loss of this structural protein around the artery permits the increased remodelling.

Further study in this area is important, as understanding the molecular mechanisms underlying enhanced outward remodelling in old mice may lead to the identification of targets to inhibit adverse media remodelling in atherosclerosis or aneurysm formation in elderly patients.

## Data Availability

The data presented in this study are available on request from the corresponding author or Principal Investigator.

## Supplementary Materials

The following supporting information can be downloaded at: https://www.mdpi.com/article/10.3390/ijms262110274/s1.
