# Supplementary material for: Augmented Flow-Induced Outward Remodelling Occurs with Ageing in Mice
Source: Int J Mol Sci. 2025 Oct 22;26(21):10274. doi: 10.3390/ijms262110274 (PMC12610229; doi:10.3390/ijms262110274)
Supplement: Supplementary file 1 [file ijms-26-10274-s001.zip › ijms-3915345-supplementary.pdf]

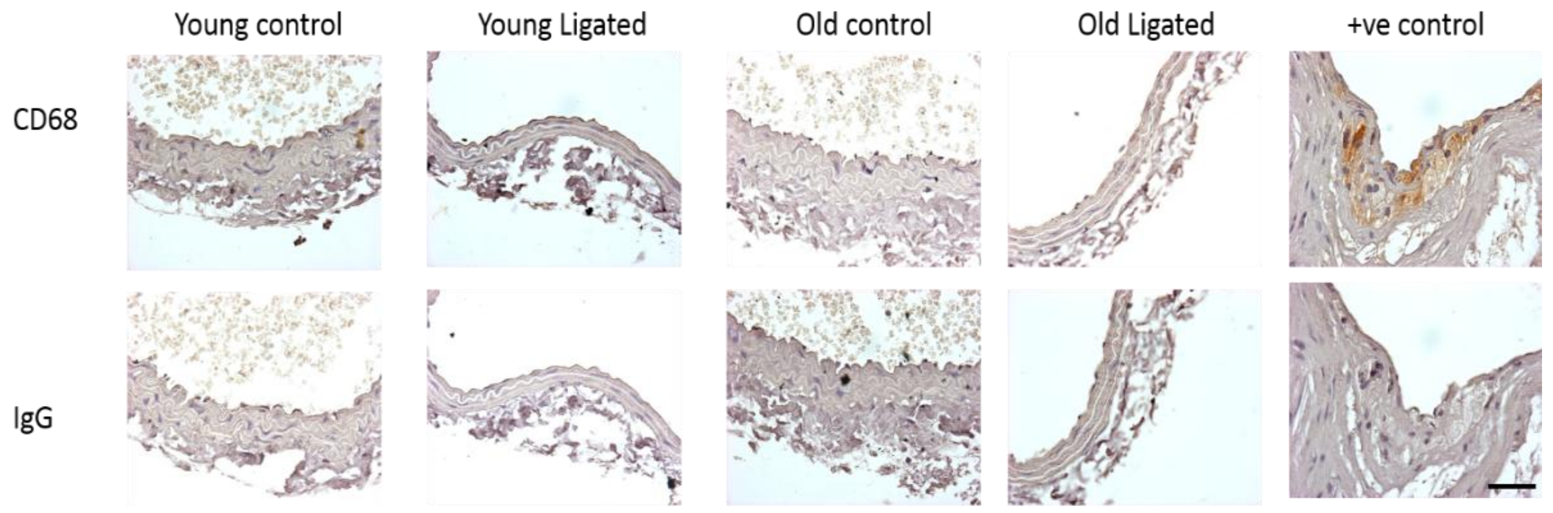

Supplementary Figure 1: Macrophages were not detected in right carotid artery sections.

Young and old mice were subjected to ligation of the left carotid artery for 21 days. Animals with unligated arteries served as controls. Macrophage content in the right carotid arteries was assessed by CD68 immunohistochemistry on sections. No CD68 macrophages were observed in right carotid arteries from any group, however, CD68 positive macrophages were observed in positive control tissue (brachiocephalic arteries from high fat diet-fed ApoE$^{-/-}$ mice). CD68 is shown in brown and nuclei are stained blue with haematoxylin. The scale bar represents 50μm and applies to all images.
